# On the Appearance of the Gums in Phthisis

**Published:** 1851-04

**Authors:** 


### ARTICLE XXI.

### *On the Appearance of the Gums in Phthisis.*

M. Fredricq published some observations in 1847, in which he stated that in phthisis a red streak is constantly to be found on the gums opposite the lower incisors, and sometimes the upper ones also. Since then he has paid great attention to the subject, and now says, that in the latter period of all chronic diseases, some time before death, a blue red streak will always be found, but that in phthisis it is one of the earliest signs. In all the cases of *phthisis*, without exception, that have come under his notice, he has met with the brick-red, or blue streak. The *brick-red* color is especially found in inflammatory phthisis, or at least when much bronchial irritation and nocturnal fever are present; while the *blue* color denotes a less active form of disease, and is oftenest in those who suffer from abundant pneumorrhagia, although by no means always indicating the probability of the occurrence of this. In a few cases a *white* line, as observed by M. Vanoye, contrasts with the general color of the mucous membrane, and those who exhibit it are usually of a marked scrofulous and cachectic habit. These streaks are of great importance in announcing early phthisis, in those cases in which doubt prevails as to whether a bronchitis or catarrh is connected with tubercle. The author has never known persons suffering from bronchitis in whom this mark was absent, afterward become the subjects of phthisis; while those in whom it was distinctly present have all afterwards furnished signs of tubercle. The deeper the color, the more rapidly does the disease proceed, while it is a good sign to find it become paler. Patients, in whom the process of softening has become temporarily arrested, and who believe themselves cured, continue to offer the blue mark, but paler than before. When the disease recommences its march, the mark becomes plainer.

Dr. Bonarden states that he has observed a blue line in *intermittent fever*. M. Fredricq has generally found it wanting, and when present it has been blue, not red, and seems to be connected with engorgement of the spleen. In *chronic abdominal* affections, there is a dirty, livid-looking streak, along the whole extent of the gums, and much broader than in phthisis. So in *menstrual molimen* there is sometimes a blue line along the free edge of the gums, which disappears when the menses comes on. It is frequently, but not constantly, observed in amenorrhea.

The following are the author's conclusions: 1. The red, blue or white streak, along the incisor gums, is only of a real semiological importance when manifested early, since it is always seen in the latter period of chronic disease. 2. It is observable as soon as the cough occurs in phthisis. 3. In aged persons, where there is retraction of the gums, it is not present. 4. Where the brick-red streak exists, it is a sure indication of tubercle; the blue one, which is common to other diseases, is also frequently so. 5. Chronic abdominal affections give rise to a livid streak along the entire gums.—M. Fredricq, *Rev. Med. Chir.*, vol. vii, pp. 139–141. From *B. & F. Med. Chir. Rev.*, Oct. 1850.

